# SW#–GPU-enabled exact alignments on genome scale

**DOI:** 10.1093/bioinformatics/btt410

**Published:** 2013-07-31

**Authors:** Matija Korpar, Mile Šikić

**Affiliations:** ^1^Faculty of Electrical Engineering and Computing, University of Zagreb, Unska 3, HR 10000 Zagreb, Croatia and ^2^Bioinformatics Institute, A*STAR, #07-01 Matrix, 138671 Singapore

## Abstract

**Summary:** We propose SW#, a new CUDA graphical processor unit-enabled and memory-efficient implementation of dynamic programming algorithm, for local alignment. It can be used as either a stand-alone application or a library. Although there are other graphical processor unit implementations of the Smith–Waterman algorithm, SW# is the only one publicly available that can produce sequence alignments on genome-wide scale. For long sequences, it is at least a few hundred times faster than a CPU version of the same algorithm.

**Availability:** Source code and installation instructions freely available for download at http://complex.zesoi.fer.hr/SW.html.

**Contact:** mile.sikic@fer.hr

**Supplementary information:** Supplementary results are available at *Bioinformatics* online.

## 1 INTRODUCTION

Sequence alignments are fundamental in bioinformatics, as they can provide information about unknown genes and proteins. They also have an important role in comparative sequence assembly (Flicek and Birney, 2009). In sequence alignments, both time and accuracy determine the successful performance of the method.

Two classic algorithms for local alignment are Smith–Waterman (Smith and Waterman, 1981) and BLAST (Altschul *et al.*, 1990). The Smith–Waterman algorithm, an exact method, aims to find the best local alignment between two sequences. It is a variation of the dynamic programming, Needleman–Wunsch algorithm (Needleman and Wunsch, 1970). BLAST, a heuristic approach, runs faster and requires less space (memory). This advantage has a high relevance for the alignment of long DNA sequences. However, BLAST does not guarantee an optimal alignment. Although original Smith–Waterman algorithm runs in quadratic time and space, the space complexity can be reduced to linear (Hirschberg, 1975; Myers and Miller, 1988). Despite the improvement in memory consumption, the Smith–Waterman algorithm runs too slowly to be useful for discovering homologous genes. Hence, newer heuristic methods have been focused on reducing computation time at the expense of accuracy. Because only exact algorithms guarantee optimal alignments, there is a lack of a method for verifying results on genome scale. In addition, popular heuristic tools for global alignment, such as MUMMER (Kurtz *et al.*, 2004) and LASTZ (Schwartz *et al.*, 2003), use dynamic programming algorithms in their final step.

The increasing popularity of using CUDA-enabled graphical processor units (GPUs) has intensified work on accelerating the Smith–Waterman algorithm. However, to the best of our knowledge, the only pairwise GPU implementation with optimized space complexity tested on the chromosome level is CUDAlign (Sandes and Melo, 2013), but it writes large amount of data to hard disk, uses a single card, and it is not publicly available.

In this article, we present our parallel implementation of the Smith–Waterman algorithm.

## 2 METHODS

The Smith–Waterman algorithm could be divided into two phases: solving and reconstruction. In the solving phase, the maximum score is calculated, whereas the optimal alignment path is derived in the reconstruction phase. The easiest approach for the reconstruction phase is by backtracking the solved matrix, but this requires quadratic space complexity. The space-efficient methods use either of the following approaches: storing each nth solved row in the matrix and using them in reconstruction phase and the divide and conquer approach (Hirschberg, 1975; Myers and Miller, 1988). CUDAlign uses the former method by storing rows on the hard disk. The latter method has only been implemented on the global alignment. The problem with local alignment includes finding the endpoints of the alignment path, for which we present our solution. Hence, our implementation consists of three phases: solving, finding and reconstruction.

**Fig. 1.**
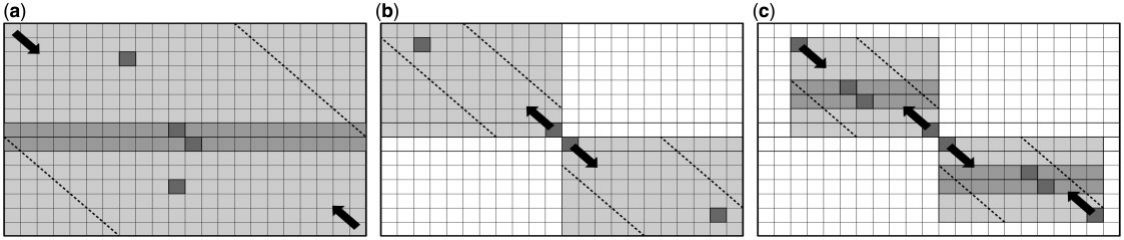
SW# done with two CUDA cards. (**a–c**) show solving, finding alignment startpoint and reconstruction phases, respectively. Gray cells represent executable area, dashed lines represent prunable area, and black arrows show direction of the execution. The best local alignment could be located in the upper part, lower part or both parts of the matrix. In the last case, the total score is the maximum sum of scores of the neighboring cells at the middle of matrix (darker gray cells). The positions of maximum scores in each phase are marked by darkest cells

In the solving phase, we used parallelization and the pruning method given by Sandes and Melo (2013). They used the wavefront method based on solving the elements on a matrix anti-diagonal at the same time. The solving matrix is divided into cell blocks. Each cell block is solved by one CUDA block and is considered a single solving unit. In this way, B*T cells can be calculated at the same time, where *B* is the number of CUDA blocks and *T* is the number of CUDA threads. The outputs of the solving phase are the total score and the endpoint of the alignment. Here, we present how this phase could be divided into two subproblems and run faster with two GPU cards (Fig. 1a). Applying the first step of the Myers–Miller algorithm calculates the endpoint and the score of the upper half as well as the endpoint and the score of the reverse bottom part. We get the middle score and the midpoint from the two calculated rows. The maximum score could be the score from the top half, bottom half or the middle score. In the latter case, we have to find both endpoints, and we can do it separately with two cards. In other cases, we have to find endpoints in either the top or bottom half of the matrix. The alignment startpoint is found by solving the modified Smith–Waterman algorithm on the reverse subsequence, which starts from the found endpoint. The differences from the original algorithm are (i) all the borders of the matrix are initialized to infinity to ensure that the alignment starts at the endpoint and (ii) the scores can drop <0 to ensure the found startpoint is connected to the endpoint. The same wavefront method of parallelization is used as in the solving phase. The cell pruning is achieved by using a banded algorithm (Ukkonen, 1985). The maximum deformation *t* (edit distance) is calculated as the ratio between the alignment score and the highest substitution score. If the lengths of the subsequences are *m* and *n*, where *m > n*, parameter *m′* can be calculated as *min(n+(n–t)/ge, m)*, where *ge* is the gap extension penalty. Padding *p* can then be calculated as ceil(0.5*(2*n–t–m′)). This observation is done by calculating the maximum score that a cell can reach and comparing it with the found score. The cell blocks that do not contain any of the diagonals between *−p* and *p+(m–n)* are pruned.

The reconstruction phase considers only the cells between the start and the endpoint (Fig. 1c). We combined the wavefront method with the modified Myers–Miller algorithm. This phase is done in parallel by both the CPU and the GPUs. For pruning, the banded algorithm is applied. The padding is calculated on the whole matrix and given to the solving halves for pruning. The maximum edit distance *t* is calculated as *max(m, n)–score*, where *m* and *n* are the lengths of the sequences between the startpoint and the endpoint, and the score is the alignment score. The Myers–Miller algorithm is applied recursively in this manner. Difference from the original algorithm is that it stops as soon as the solving submatrix size drops below defined boundaries. When this size is small enough, GPU part sends it to the CPU part. As the scores of the submatrices are known, the CPU part performs the banded algorithm with the backtracking. Finally, alignments of the submatrices are joined in the complete alignment.

## 3 RESULTS

Application run times are displayed in Table 1. Measurements are done using two different GPU cards and CPU Intel® Quad Processor Q6600. The GTX 570 card on which we performed measurements is slightly more capable than GTX 560 used in Sandes and Melo (2013). We present the result achieved with a maximum of two GPU cores. Compared with the presented results for CUDAlign, the scores were identical. SW# is slower only for very long alignments on single GPU card. However, as opposed to CUDAlign, it does not use any additional disk space, can use multiple cards and is publicly available. Moreover, the global and semi-global dynamic programming algorithms are implemented. SW# could also be used as a library in all methods that use exact alignment of long sequences in one of the steps. Supported operating systems are Windows, Linux and Mac OS.

**Table 1.** SW# and CUDAlign run time comparison for different NVIDIA GPU cards and sequence lengths

Sequences size	CPU	GTX 560 CUDAlign^a^	GTX 570 SW#	GTX 690^b^ SW#
2 × 172 Kb	660 s	2.1 s	1.5 s	2.6 s
0.5 × 0.5 Mb	9090 s	11.8 s	9.4 s	5.8 s
3.1 × 3.3 Mb	—	367 s	296 s	119 s
59 × 24 Mb	—	47123 s	40359 s	16263 s
33 × 47 Mb	—	30369 s	59228 s	23614 s

*Note*: CPU results are presented only for shorter sequences.

^a^CUDAlign results are taken from the original article (Sandes and Melo, 2013).

^b^Dual GPU card.

## Supplementary Material

Supplementary Data
