# Supplementary material for: SW#–GPU-enabled exact alignments on genome scale
Source: Bioinformatics. 2013 Jul 31;29(19):2494–5. doi: 10.1093/bioinformatics/btt410 (PMC3777108; doi:10.1093/bioinformatics/btt410)
Supplement: Supplementary Data [file supp_29_19_2494__index.html]

SW# - GPU enabled exact alignments on genome scale — SW#–GPU-enabled exact alignments on genome scale — SW#–GPU-enabled exact alignments on genome scale — Supplementary Data 

# SW#–GPU-enabled exact alignments on genome scale

## 

files

**Files in this Data Supplement:**

- Supplementary Data - doc file
