# Supplementary material for: SW#–GPU-enabled exact alignments on genome scale
Source: Bioinformatics. 2013 Jul 31;29(19):2494–5. doi: 10.1093/bioinformatics/btt410 (PMC3777108; doi:10.1093/bioinformatics/btt410)
Supplement: Supplementary Data [file supp_btt410_Supplementary_Results.doc]

| Supplementary data  SW# - GPU enabled exact alignments on genome scale  Korpar, Matija^1^, Šikić, Mile^1,2*^  ^1^ University of Zagreb, Faculty of Electrical Engineering and Computing, Unska 3, HR 10000 Zagreb, Croatia  ^2^ Bioinformatics Institute, A*STAR, 30 Biopolis Street, #07-01 Matrix, 138671 Singapore |
| --- |

RESULTS

Supplementary table 1. Run time comparison for implementations of the Smith-Waterman algorithm on different computing platforms. Results are presented for 4 different implementations: a standard CPU implementation of the algorithm, SSW (Zha*o et a*l., 2012) an implementation which uses the Single-Instruction Multiple-Data (SIMD) instructions to parallelize the algorithm at the instruction level and two GPU implementations CUDAlign (Sandes and Melo, 2013) and SW#. SSW algorithm does only the solving part of the Smith-Waterman algorithm. SW# measurements are done on three versions of NVIDIA GPU cards, covering past three CUDA architectures. Measurements on GTX690 card are made with using two GPUs and a single one to demonstrate scalability. Both of the tested CPU versions are too slow on bigger inputs or simply require too much memory.

| **Sequence sizes** | **Standard CPU implementation**  **Intel® Quad Q6600** | **SSW**  **Intel ®Sandy Bridge i7** | **CUDAlign GTX 560** | **SW#**  **Tesla C10*** | **SW#**  **GTX 570** | **SW#**  **GTX 690** | **SW#**  **GTX 690*** |
| --- | --- | --- | --- | --- | --- | --- | --- |
| **2Kb×172Kb** | 660s | 7.51s | 2.1s | 1.66s | 1.5s | 2.87s | 2.6s |
| **0.5Mb×0.5Mb** | 9090s | 77.42s | 11.8s | 10.85s | 9.4s | 9.00s | 5.8s |
| **3.1Mb×3.3Mb** | - | 9869.78s | 367s | 316s | 296s | 234s | 119s |
| **59Mb×24Mb** | - | - | 47123s | 52090s | 40359s | 31449s | 16263s |
| **33Mb×47Mb** | - | - | 30369s | 73143s | 59228s | 44690s | 23614s |

*dual GPU cards

Supplementary table 2. Run time comparison for implementations of SW# and a FPGA implementation (Zhan*g et a*l., 2007). Only the solving parts of the algorithm were compared and times do not include architecture initialization or any IO operations. As it can be seen the bigger the sequences are the faster SW# will be. FPGA version cannot handle input sequences larger than 65536 bases, on the other hand SW# can handle input sequences up to 110 Mb on NVIDIA GTX 570 (1,280 MB RAM). To the best of our knowledge the implementation of Zhang et al is the FPGA implementation that can handle longest sequences. As shown in (Hosny and Shedeed, 2011) only GPU implementation can run alignment of sequences longer than 10 Mb in reasonable time.

| **Sequence sizes (base pairs)** | **SW FPGA** | **GTX 570 SW#** |
| --- | --- | --- |
| **256×256** | 0.000226s | 0.001s |
| **512×512** | 0.000374s | 0.003s |
| **1024×1024** | 0.000472s | 0.004s |
| **2048×2048** | 0.000898s | 0.006s |
| **4096×4096** | 0.001781s | 0.009s |
| **8192×8192** | 0.005s | 0.014s |
| **16384×16384** | 0.014s | 0.026s |
| **32768×32768** | 0.049s | 0.058s |
| **65536×65536** | 0.182s | 0.144s |

SW# provides both a library for sequences alignment as well as standalone executables. Main goal of SW# software is to provide fast and stable library for usage with other alignment software. Library includes the parallel version of local alignment (Smith and Waterman, 1981), global alignment (Needleman and Wunsch, 1970) and semi-global alignment. Complete library is written in C and CUDA and is available on Windows, Linux and Mac OS. SW# is publicly available on <https://sourceforge.net/projects/swsharp/>.

References

Hosny,A. and Shedeed,H. (2011) An efficient solution for aligning huge DNA sequences. *International Journal of Computer Applications*, **32**, 1–8.

Needleman,S.B. and Wunsch,C.D. (1970) A general method applicable to the search for similarities in the amino acid sequence of two proteins. *Journal of molecular biology*, **48**, 443–53.

Sandes,E.F.D.O. and Melo,A.C.M.A. De (2013) Retrieving Smith-Waterman Alignments with Optimizations for Megabase Biological Sequences using GPU. *IEEE Transactions on Parallel and Distributed Systems*, **24**, 1009–1021.

Smith,T.F. and Waterman,M.S. (1981) Identification of common molecular subsequences. *Journal of Molecular Biology*, **147**, 195–197.

Zhang,P. *et al.* (2007) Implementation of the Smith-Waterman algorithm on a reconfigurable supercomputing platform. In, *Proceedings of the 1st international workshop on High-performance reconfigurable computing technology and applications held in conjunction with SC07 - HPRCTA  ’07*. ACM Press, New York, New York, USA, p. 39.

Zhao,M. *et al.* (2012) SSW Library: An SIMD Smith-Waterman C/C++ Library for Use in Genomic Applications. *arXiv preprint arXiv:1208.6350*, **00**, 1–3.
